# Lernen für die Prävention: Alkoholkonsum aus verschiedenen Perspektiven und in unterschiedlichen Bevölkerungsgruppen und Settings

**DOI:** 10.1007/s00103-021-03344-7

**Published:** 2021-06-08

**Authors:** Freia De Bock, Uwe Koch-Gromus

**Affiliations:** 1 Abteilung 2 Effektivität und Effizienz der gesundheitlichen Aufklärung, Bundeszentrale für gesundheitliche Aufklärung (BZgA), Maarweg 149–161, 50825 Köln, Deutschland; 2 Institut und Poliklinik für Medizinische Psychologie, Universitätsklinikum Hamburg-Eppendorf, Martinistraße 52, 20246 Hamburg, Deutschland

Alkohol ist weltweit die am weitesten verbreitete psychoaktive Substanz. Deutschland ist mit Durchschnittskonsumwerten von 11,8 l pro Kopf und Jahr (2008–2010, WHO [World Health Organization]) eines der Hochkonsumländer. Als weitverbreitetes Konsumgut gehört Alkohol in der alternden Gesellschaft mit vielen relativ wohlhabenden älteren Menschen zur Alltagskultur, in Form des „Weinchens“ am Abend als Entspannungsritual, was Alkohol zunehmend salonfähig bzw. sogar zum Symbol von „Arriviertsein“ macht. Aber auch Jugendliche und junge Erwachsene sind nicht abgeneigt. Sie präsentieren sich z. B. volltrunken auf Partyfotos in sozialen Medien. (Wie die kontakteinschränkenden Maßnahmen in der COVID(„coronavirus disease“)-19-Pandemie die Verbreitung von Alkoholkonsum verändern, ist noch nicht abschließend geklärt.)

Die Prävalenz, Qualität und Form des Alkoholkonsums sowie die Verbreitung der Alkoholsucht unterscheiden sich nach Bevölkerungsgruppen und verändern sich über die Zeit. So ist beispielsweise unter Erwachsenen bei beiden Geschlechtern ein fallender Trend der Alkoholkonsumprävalenz zu beobachten, wobei die Prävalenz bei Männern höher ist als bei Frauen. Zudem besteht bei Erwachsenen ein Zusammenhang zwischen Sozialstatus und Häufigkeit des Alkoholkonsums, mit höherem Konsum in der Gruppe mit höherem Sozialstatus. Auch bei Jugendlichen ist der Alkoholkonsum in den letzten Jahren insgesamt zwar rückläufig, bestimmte Untergruppen von Jugendlichen sind jedoch weiterhin gefährdet, z. B. durch Rauschtrinken.

Ein übermäßiger Alkoholkonsum ist in der Altersgruppe 15–49 Jahre für nahezu 10 % aller Todesfälle weltweit verantwortlich. Langfristig bestehen erhöhte gesundheitliche Risiken, u. a. für Krebserkrankungen, Leber- und kardiovaskuläre Erkrankungen, Diabetes und beispielsweise auch Veränderungen im Sexualhormonstoffwechsel. Aber nicht nur das konsumierende Individuum selbst setzt sich Risiken aus, auch generationenübergreifende Effekte von Alkoholkonsum sind schwerwiegend. Schon mäßiger Alkoholkonsum während der Schwangerschaft kann zu einer Schädigung des Fötus führen, in der Maximalform zur Diagnose des fetalen Alkoholsyndroms mit schweren Herzfehlern und geistiger Behinderung.

Systematische Evidenzsynthesen der letzten Jahre zeigen, dass es keinen sicheren Alkoholkonsum gibt: Die Autoren der im *Lancet* veröffentlichten systematischen Übersicht aus dem Jahr 2018 schlussfolgerten: „Our results show that the safest level of drinking is none“. Auch wenn in Deutschland bisher lediglich das Gesundheitsziel „Alkoholkonsum reduzieren“ konsentiert ist, muss der Paradigmenwechsel im internationalen Diskurs ernst genommen werden. Eine möglichst frühe und umfassende Prävention des Alkoholkonsums mit effektiven, bevölkerungsweiten Maßnahmen ist dafür notwendig.

Das vorliegende Heft beschreibt, in welchem kulturellen, gesellschaftlichen und epidemiologischen Kontext sich Präventionsmaßnahmen zum Alkoholkonsum bewähren müssen, stellt konkrete verhaltenspräventive und verhältnispräventive Ansätze vor, erörtert die Rolle von lebensweltbezogenen Veränderungen, beschreibt die Möglichkeiten des Präventionsgesetzes und gibt einen Überblick über die Evidenz zu konkreten Maßnahmen aus dem nationalen und internationalen Umfeld.

Der erste Beitrag von *A. Heinz* und *L. S. Daedelow* fokussiert die Rolle des Alkohols als Kulturgut in westlichen Gesellschaften. Neben dem Überblick über die historische Entwicklung des Alkoholkonsums wird die soziokulturelle Diversität in westlichen Gesellschaften beleuchtet: der Umgang mit Alkohol und die Einschätzung seines Gebrauchs sowie deren Relevanz für klinische Interventionen.

*L. Kraus et al.* analysieren auf der Grundlage der Erhebungen der Jahre 1995 bis 2018 mit dem Instrument des Epidemiologischen Suchtsurveys (ESA) zeitliche Trends des riskanten Konsums und des episodischen Rauschtrinkens nach Altersgruppen und Geschlecht im Erwachsenenalter. Geprüft wird darüber hinaus, wie sich mögliche Entwicklungstrends in den Altersgruppen darstellen.

Inhaltlich an das Thema Rauschtrinken knüpft die Untersuchung von *B. Orth *und* C. Merkel *an*.* Sie analysieren diese Form des Alkoholmissbrauchs bei Jugendlichen und jungen Erwachsenen, bei denen dieses häufig vorkommt und die hirnbiologisch vulnerabler sind als Erwachsene. Die Analysen berücksichtigen auch Bildungsniveau und Migrationshintergrund.

Alkoholprävention ist ein zentrales Thema der Arbeit der Bundeszentrale für gesundheitliche Aufklärung. *T. Schwarz *und *M. Goecke* stellen in ihrem Überblicksartikel über verschiedene BZgA-gestützte Kampagnen die aktuellen bundesweiten Interventionen innerhalb der Suchtprävention dar. Diese zeichnen sich als Mehrebenenkampagnen aus, die in vielfältiger Form im Sinne von integrierten Strategien miteinander verbunden sind. Ein begleitendes Monitoring in Form von Repräsentativbefragungen ergänzt die Gesamtkonzeption.

Neben bundesweiten kommunikativen Maßnahmen kann Prävention von Alkoholmissbrauch auch in den Lebenswelten von Menschen vor Ort ansetzen*. T. Praßer, H.-J. Hallmann* und *M. Goecke* beschreiben die unterschiedlichen Strategien und Organisationsstrukturen der kommunalen Alkoholprävention. Der Beitrag zeigt auch die Probleme auf, mit denen Kommunen bei der Umsetzung alkoholpräventiver Interventionen konfrontiert sind. Die Analyse legt zudem offen, dass es auf der kommunalen Ebene einer verbesserten Qualifizierung von Fachkräften und eines schnelleren Transfers von Forschungsergebnissen in die Versorgungspraxis bedarf.

Der Beitrag von *E. Wienemann *und* A. Wartmann *bietet einen Überblick über aktuelle Suchtpräventionsprogramme im betrieblichen Setting. Die betriebliche Alkohol- und Suchtprävention als Teil des betrieblichen Gesundheitsmanagements wirken dort besonders nachhaltig, wo das Programm auf der Basis einer Betriebsvereinbarung verbindlich umgesetzt wird.

Im anschließenden Beitrag von *N. Döring *und *C. Holz *wird im Rahmen einer Social-Media-Analyse untersucht, welche deutschsprachigen alkoholbezogenen Kanäle auf Plattformen wie Facebook und Instagram eine große Reichweite haben. Darüber hinaus werden die Nutzerkommentare des reichweitenstärksten Social-Media-Kanals für Alkoholprävention in Deutschland, der Facebookseite der BZgA-Jugendkampagne „Alkohol? Kenn dein Limit.“, analysiert. In den sozialen Medien dominiert gegenwärtig die Alkoholverherrlichung. Hier bedarf es neuer Strategien, die dazu führen sollten, dass Botschaften aus der Suchtprävention stärker an Bedeutung gewinnen.

*M. Morgenstern, J. Hansen* und *R. Hanewinkel *untersuchen im Rahmen einer bundesweiten und längsschnittlichen Untersuchung bei mehr als 2500 Schülern/innen der 6. bis 8. Jahrgangsstufe, ob das Probieren von Alkohol ein unabhängiger Prädiktor für einen frühen Einstieg in das Rauschtrinken darstellt. Die Untersuchungsergebnisse bestätigen den unterstellten Zusammenhang. Die Autoren/innen sehen einen Bedarf an weiteren Studien zur Aufklärung bestehender Kausalitäten.

*S. Diestelkamp, A.-L. Schulz* und *R. Thomasius *berichten Ergebnisse eines narrativen Reviews zu technologiebasierten Interventionen und Frühinterventionen zur Prävention des riskanten Alkoholkonsums bei Kindern und Jugendlichen. Die Analyse zeigt das erhebliche Potenzial technologiebasierter Präventionsmaßnahmen in einer Vielzahl von Settings. Für den Erwachsenenbereich sind die technologiebasierten Maßnahmen, die häufig über interaktive Webseiten, Apps oder SMS-Nachrichten umgesetzt werden, in ihrer Wirksamkeit belegt. Für den Bereich Kinder und Jugendlicher bedarf es weiterer Untersuchungen.

Die drei nachfolgenden Kurzbeiträge stellen Best-Practice-Modelle der Suchtprävention vor. Zunächst berichten *R. Hanewinkel, D. Hammes* und *B. Isensee* über ein Angebot für Jugendliche in der späten Adoleszenz, das unter dem Slogan „Klar bleiben“ für Schüler/innen der 10. Jahrgangsstufe entwickelt wurde. Erste Ergebnisse der wenig aufwendigen Intervention von „Klar bleiben“ beschreiben die Autoren/innen als ermutigend.

Der zweite Kurzbeitrag von *P. Eichin* und *H. Kuttler* bezieht sich auf das Alkoholpräventionsprogramm „HaLT – Hart am LimiT“. Hinter dem bundesweit angelegten Programm stehen die Villa Schöpflin gGmbH, das Bundesnetzwerk HaLT und eine Kooperation mit dem GKV-Bündnis für Gesundheit. Die Konzeption ist auf eine komplexe bundesweite Implementierung des Programms über Landeskoordinatoren ausgerichtet. Im Artikel werden die Neuentwicklungen des schon länger erfolgreich laufenden Programms thematisiert.

Das dritte Best-Practice-Beispiel für Suchtprävention „Aktion Glasklar“ wird von *B. Isensee* und *R. Hanewinkel* dargestellt. Es handelt sich um eine schon länger laufende Maßnahme, in der Jugendliche sach- und altersgerecht mit multimodalen Zugängen über Alkohol aufgeklärt und zu einer kritischen Reflexion angeregt werden sollen. Als Mediatoren/innen dienen Bezugspersonen wie Lehrkräfte und Jugendgruppenleiter/innen. Die Evaluation belegt die grundsätzliche Wirksamkeit von „Aktion Glasklar“ auf das Risiko des Rauschtrinkens.

*A. Bühler, J. Thrul *und *E. Gomes de Matos* stellen die Ergebnisse einer von der Bundeszentrale für gesundheitliche Aufklärung (BZgA) im Jahr 2020 in Auftrag gegebenen Expertise zur Suchtprävention dar. Diese Expertise zieht eine Reihe von alkoholbezogenen Schlussfolgerungen. Insbesondere für die Präventionsfelder Familie, Schule, Medien und Kommune konnten wirksame Ansätze identifiziert werden.

Der letzte Beitrag von *J. E. Moder et al.* greift einen sehr speziellen, aber hoch relevanten Teilaspekt auf, nämlich die fetalen Alkoholspektrumstörungen. Ein Problem bei diesen somatischen und kognitiven Entwicklungsstörungen, die aufgrund eines fortgesetzten Alkoholkonsums der Mutter während der Schwangerschaft entstehen, ist die Tatsache, dass sie häufig nicht diagnostiziert oder fehldiagnostiziert werden. Die Autoren/innen fordern neue Präventionskonzepte und entsprechende gesundheitspolitische Maßnahmen.

Wir glauben, dass in diesem Heft durch die ausgewählten Beiträge das Thema Alkoholprävention sehr differenziert betrachtet wird und dass ein Themenheft vorliegt, das viel Stoff für Diskussionen im wissenschaftlichen wie im sozialen Feld liefert.

Wir wünschen Ihnen, liebe Leserin und lieber Leser, eine spannende Lektüre.

Prof. Dr. Freia De Bock und Prof. Dr. Dr. Uwe Koch-Gromus

